# Air Pollution: The Plaque of the Matter

**Published:** 2006-04

**Authors:** Rebecca Clay Haynes

Particulate matter measuring less than 2.5 microns (PM_2.5_) has been widely linked to heart disease. These tiny particles of dust, soot, and smoke accompany emissions from power plants and vehicle exhaust, and lead to an estimated 60,000 premature deaths per year in the United States. EPA standards limit average human exposure of PM_2.5_ to a maximum of 15 micrograms per cubic meter. Now a study published in the 21 December 2005 issue of *JAMA* shows that long-term exposure to PM_2.5_ even at levels within federal standards accelerates the development of atherosclerosis in laboratory mice by increasing plaque development, especially when the mice are also fed a high-fat diet.

Mice do not naturally develop plaque, a fatty deposit on the inner lining of arteries; the 28 mice used in the study were genetically modified to do so. The animals were divided into two subgroups, one fed a normal diet, the other a high-fat diet. Those subgroups were then divided again, with half breathing air containing concentrated PM_2.5_ at levels equivalent to 15.2 micrograms per cubic meter and the other half breathing particle-free filtered air. The air pollution group was exposed for six hours per day, five days per week, for six months.

Researchers found measurable changes in the extent and severity of plaque formation in the aorta as well as artery inflammation and reduced function of the arterial lining. In animals that breathed polluted air and ate a high-fat diet, 41.5% of the arterial interior measured was filled with plaque, compared to 13.2% in animals that breathed filtered air and ate a normal diet.

In humans, high plaque levels can lead to heart attacks and strokes. “These results can be very applicable to the human population, especially in urban environments,” says coauthor Lung Chi Chen, an associate professor of environmental medicine at the New York University School of Medicine. “We found that the combination of air pollution and diet had a dramatic effect on plaque formation, leading to inflammation and heart disease.”

“It was surprising in this study to see the impact of exposure to a relatively low PM concentration on plaque development,” says Kevin Dreher, a principal investigator studying the cardiovascular effects of air pollution within the EPA’s National Health and Environmental Effects Research Laboratory. “While both dietary groups developed plaque when exposed to polluted air, the high-fat diet led to more consistent and statistically significant increase alterations of plaque development. Fat in the diet appears to be an important effect modifier when coupled with exposure to air particulate pollution.”

Chen and his group are now gathering results on heart rate and blood pressure changes in these same mice. In ongoing studies, they are also examining specific mechanisms that could link air pollution, diet, and heart disease. In particular, they are trying to better define which components in PM_2.5_ most likely promote plaque formation and atherosclerosis.

